# *Ginkgo biloba* extract alleviates fatty liver hemorrhagic syndrome in laying hens via reshaping gut microbiota

**DOI:** 10.1186/s40104-023-00900-w

**Published:** 2023-08-03

**Authors:** Xinyue Yang, Depeng Li, Meihong Zhang, Yuqing Feng, Xiaolu Jin, Dan Liu, Yuming Guo, Yongfei Hu

**Affiliations:** State Key Laboratory of Animal Nutrition, College of Animal Science and Technology, China Agricultural University, Beijing, 100193 China

**Keywords:** Antioxidation, Fecal microbiota transplantation, FLHS, *Ginkgo biloba *extract, Inflammation, *Megasphaera*

## Abstract

**Background:**

*Ginkgo biloba *extract (GBE) is evidenced to be effective in the prevention and alleviation of metabolic disorders, including obesity, diabetes and fatty liver disease. However, the role of GBE in alleviating fatty liver hemorrhagic syndrome (FLHS) in laying hens and the underlying mechanisms remain to be elucidated. Here, we investigated the effects of GBE on relieving FLHS with an emphasis on the modulatory role of GBE in chicken gut microbiota.

**Results:**

The results showed that GBE treatment ameliorated biochemical blood indicators in high-fat diet (HFD)-induced FLHS laying hen model by decreasing the levels of TG, TC, ALT and ALP. The lipid accumulation and pathological score of liver were also relieved after GBE treatment. Moreover, GBE treatment enhanced the antioxidant activity of liver and serum by increasing GSH, SOD, T-AOC, GSH-PX and reducing MDA, and downregulated the expression of genes related to lipid synthesis (*FAS*, *LXRα*, *GPAT1*, *PPARγ* and *ChREBP1*) and inflammatory cytokines (*TNF-α, IL-6, TLR4* and *NF-κB*) in the liver. Microbial profiling analysis revealed that GBE treatment reshaped the HFD-perturbed gut microbiota, particularly elevated the abundance of *Megasphaera* in the cecum. Meanwhile, targeted metabolomic analysis of SCFAs revealed that GBE treatment significantly promoted the production of total SCFAs, acetate and propionate, which were positively correlated with the GBE-enriched gut microbiota. Finally, we confirmed that the GBE-altered gut microbiota was sufficient to alleviate FLHS by fecal microbiota transplantation (FMT).

**Conclusions:**

We provided evidence that GBE alleviated FLHS in HFD-induced laying hens through reshaping the composition of gut microbiota. Our findings shed light on mechanism underlying the anti-FLHS efficacy of GBE and lay foundations for future use of GBE as additive to prevent and control FLHS in laying hen industry.

## Background

Fatty liver hemorrhagic syndrome (FLHS), a kind of nutritional metabolic disease in laying hens, is characterized by excessive accumulation of hepatic and abdominal fat, hepatic steatosis, liver rupture, inflammatory response and hepatorrhagia [1, 2]. FLHS frequently occurs in commercial caged high-production and over conditioned laying hens, accounting for 40%–70% of hen mortality [3]. Moreover, FLHS can cause sharp drop of egg production rate and a shortened egg production peak in hen flocks. Therefore, FLHS has been regarded as a “silent killer” for both laying hens and producers [1]. The pathogenesis of FLHS is poorly understood but has a great similarity to metabolic dysfunction-associated fatty liver disease (MAFLD) in human beings [4], and the common pathologic feature of FLHS and MAFLD is excess hepatic lipid deposition. It is suggested that the FLHS laying hen can be used as an ideal animal model for investigating MAFLD and hepatic steatosis in humans [4–6].

Studies have shown that dietary ingredients, stress, hormones, genetics, environment, inflammation, lipid metabolism disorder, oxidative stress, autophagy, gut microbiota and other pathogenic factors are related to FLHS [7]. Recently, the importance of gut microbiota in the progression of FLHS has been widely concerned [2, 4]. It is indicated that FLHS was linked with the laying hen cecal microbial dysbiosis, and the change in the abundances of Firmicutes and Bacteroidetes was closely associated with the severity of fibrosis and nonalcoholic steatohepatitis [4]. There are also studies showed that the non-hepatic steatosis group had more beneficial bacteria, while the hepatic steatosis group had more harmful bacteria [2, 8]. Additionally, treatment with *Akkermansia muciniphila* or the decoction of *Abrus cantoniensis Hance* efficiently reshaped the high-fat diet (HFD)-perturbed intestinal microbiota and thus exerted anti-FLHS effects in laying hens [2, 9]. The changes of gut microbiota were also found related to the host oxidative stress and inflammatory response, both of which are regarded as factors that contribute to the development and progression of FLHS. A recent study showed that the interaction between intestinal epithelial cells and some gut commensal bacteria induces rapid production of reactive oxygen species (ROS) in host cells [10], and the mechanism by which ROS promotes the progression of MAFLD may be related to abnormal oxidative stress markers and dysregulated redox signaling [11]. Interestingly, evidence suggest that alterations of gut microbiota regulate the metabolism of the major intracelluar antioxidant (glutathione (GSH)) in the host organism [12], as well as affect Nox2 (oxidative stress markers) activation and redox signaling [10, 13]. As for inflammation, studies have shown that the altered gut microbiota (e.g., reduction of *bifidobacteria*) cause an increase in plasma lipopolysaccharide levels, which lead to increases in proinflammatory cytokines [14]. However, the use of probiotic bacteria alleviates liver inflammation by inhibiting the production of pro-inflammatory cytokines and improving liver function [15, 16]. Additionally, microbiota-derived metabolites (e.g., short-chain fatty acids (SCFAs)) are evidenced to have anti-MAFLD properties. For example, *Astragalus polysaccharides*-enriched *Desulfovibrio vulgaris* attenuates MAFLD via generating acetic acid [17]. Therefore, reconstructing the micro-ecological system via prebiotics, probiotics, or microbial products is a promising method to attenuate FLHS [18].

*Ginkgo biloba *extract (GBE) mainly contains 24% flavonoids glycosides (quercetin, kaempferol, isorhamnetin etc.), 6% terpenoids (in which 3.1% are ginkgolides A, B, C and J and 2.9% is bilobalide), and 5%–10% organic acids [19]. GBE is a traditional Chinese medicine known for its various biological properties, such as antineoplastic, hepatoprotective, vasodilatory, antiedematogenic, antioxidant, anti-inflammatory, antiplatelet, immune modulation and metabolic regulation [20, 21]. Flavonoids and terpenoids are suggested to be the pharmacologically active components of GBE [22, 23]. Considerable evidence has shown that GBE is effective in the prevention and alleviation of atherosclerosis [24], cardiovascular diseases [25, 26], obesity [27, 28], MAFLD [29], neurological diseases [30], among others. Although the beneficial role of GBE can be partially attributed to its antioxidant activity, recent findings strongly suggested that GBE and its components can interact with gut microbiota to perform biological functions. For example, GBE has been shown to ameliorate hypercholesterolemia, systemic inflammation and atherosclerosis, and these effects are associated with rebalancing gut flora and microbial metabolism [24]. Another study showed that polysaccharide from *Ginkgo biloba *leaves reduced stress-induced depression and reversed depression-associated gut dysbiosis while increasing the abundance of *Lactobacillus* species [30]. Additionally, bilobalide is an extract of *Ginkgo biloba* that has been shown to attenuate the severity of ulcerative colitis by inhibiting inflammation, promoting the repair of the intestinal epithelial barrier and remodeling the intestinal microbial communities, especially by promoting the proliferation of probiotic species including *Lactobacillus* [31]*.* Similarly, supplementation with kaempferol, a flavonoid, offset the imbalance of intestinal flora associated with obesity and inflammatory [32].

Thus far, limited evidence has been provided on the role of GBE in alleviating FLHS in laying hens, and whether and how GBE modulates the chicken gut microbiota to exert the anti-FLHS effect are still needed to be answered. In the current study, we utilized HFD induced FLHS laying hens as a model to determine the effect of GBE on attenuating FLHS. We further performed FMT experiment to investigate the protective effect of the GBE-altered gut microbiota on FLHS. We demonstrated that GBE effectively alleviated FLHS in the HFD-induced laying hen model, and this effect could be largely due to the influence of GBE on the laying hen gut microbiota structure and function. Our study confirmed the therapeutic potential of GBE for FLHS in laying hens, and revealed the underlying gut microbiota-mediated mechanism.

## Methods

### Animals and experimental design

#### Ethics statement

All animal experimental procedures were conducted according to guidelines of the Institutional Studies Animal Care and Use Committee of China Agricultural University (Beijing, China). The study was approved by the Ethics Committees of Laboratory Animal Center of China Agricultural University (Beijing, China) (permit AW13501202-2–2).

#### Chicken raising

HY-Line gray commercial laying hens were purchased and housed in Zhuozhou breeding base of China Agricultural University (Zhuozhou, China). All hens were housed in stainless-steel ladder cages (45 cm × 45 cm × 45 cm) and in a controlled room, in which temperature and relative humidity was maintained at 26 ± 2 °C and 50% ± 10%, respectively. The photoperiod was a 16 h light/8 h dark with a dark cycle encompassing 22:00 to 6:00. All the birds were allowed free access to water and food. All the birds were subjected to an acclimatization period of one week before formal test, during which they were fed a normal diet (ND), and meet the NRC (1994) [33] and the management manual of Hy-Line gray laying hens’ recommendations (https://www.hyline.com/literature/sonia). The ingredients and nutrient levels of ND are presented in Table 1.

**Table 1:** Composition and nutrient level of experimental diets

Items	Normal diet	High-energy low-protein diet
Ingredient, %		
Corn	62.05	62.05
Soybean meal	23.43	14.91
Limestone	8.47	8.47
Wheat bran	2.46	4
Dicalcium phosphate	1.25	1.34
Zeolite	0.56	0.724
Salt	0.35	0.35
Mineral premix^1^	0.25	0.25
Vitamin premix^2^	0.08	0.08
Santoquin	0.03	0.03
*DL*-Methionine	0.09	0.146
*L*-Lysine HCl (78%)	/	0.32
Choline chloride (50%)	0.08	0.08
Soybean oil	0.9	/
Lard	/	7.25
Total	100	100
Nutrient levels^3^		
ME, Mcal/kg	2.7	3.09
Crude protein, %	16.501	13
Crude fat, %	3.501	9.656
Ca, %	3.5	3.5
Available P, %	0.333	0.333
Met, %	0.347	0.347
Lys, %	0.859	0.859

^1^The mineral premix provided the followings per kg of diets: Mn 60 mg, Fe 80 mg, Cu 8 mg, I 1.2 mg, Se 0.3 mg

^2^The vitamin premix provided the followings per kg of diets: vitamin A 12,500 IU, vitamin D_3_ 32,500 IU, vitamin E 18.75 mg, vitamin K_3_ 2.65 mg, vitamin B_1_ 2 mg, vitamin B_2_ 6 mg, vitamin B_12_ 0.025 mg, pantothenic acid 12 mg, niacin 50 mg, folic acid 1.25 mg, biotin 0.325 mg

^3^Nutrient levels were calculated values, which based on the analyzed data for the experimental diets

#### Exp. 1: Effect of GBE on FLHS laying hens induced by HFD

A total of 135 24-week-old Hy-Line gray commercial laying hens of similar weight were raised in ladder cages with three bird each cage. The hens were then randomly divided into three groups, with 15 replicates per group and three hens per replicate. The control group continued receiving ND while the model group was fed high-energy low-protein diet (HFD) throughout the experiment to induce FLHS [2, 34]. Diet compositions and nutrient levels of HFD are shown in Table 1. In addition, on the basis of the HFD, GBE was supplemented at the concentration of 1,200 mg/kg diet. GBE was purchased from Xuzhou Hengkai Ginkgo Products Co., Ltd. (Xuzhou, China). GBE were first mixed with the premix and then mixed with other ingredients by the principle of step-by-step premixing and stored at 4 °C before feeding. Diets were prepared every two weeks during the experiment to ensure the quality of diets. All diets were prepared in the feed laboratory of Zhuozhou breeding base of China Agricultural University. The formal experimental period was from 25 to 40 weeks of age (total 16 weeks).

The day before the end of the experiment, all laying hens were fasted for 8 h, but water was offered ad libitum. Then ten laying hens (one hen per cage) were randomly selected and marked from 15 replicates for each treatment group, and blood was taken aseptically from the wing vein using 5-mL vacuum blood tubes. After the serum was separated naturally, it was centrifuged at 3,000 × *g* for 10 min to separate out the serum. Pure serum samples were aspirated by pipette, stored in 1.5-mL centrifuge tubes at −80 °C for subsequent test. At the end of the experiment, marked laying hens were euthanized via cutting the jugular vein. The liver was imaged and weighed, and approximately 1 cm of liver tissue was taken and fixed in 4% paraformaldehyde for histopathological evaluation. Three small pieces of liver tissue were taken, divided them into three centrifuge tubes, snap-frozen in liquid nitrogen and stored at −80 °C. One tube is used to measure the fat content of the liver, one tube is used to measure the antioxidant activity of the liver, and the other tube is used for mRNA analysis. The heart and abdominal fat were weighed. Cecal and ileal contents were immediately transferred to liquid nitrogen and kept in a −80 °C freezer for further analysis.

#### Exp. 2: Fecal microbiota transplantation

##### Preparation of fecal bacterial suspension for FMT

We prepared fecal bacterial suspensions as previously described [35–37]. Fresh feces from the hens of HFD_GBE group were immediately collected and immersed in liquid nitrogen within 2 h after defecation. Then, the frozen feces were transported on ice to a laboratory for preparation of fecal bacteria suspension. Briefly, 50 g fecal samples are diluted with 250 mL sterile saline and homogenized by hot plate magnetic stirrer (IT-09A5, Shanghai Yiheng Technology Co., Ltd., Shanghai, China). Then, the slurry was filtered one by one through stainless steel sieves of 2, 1, 0.5, 0.25 mmol/L to eliminate the undigested and small particulate matter in the fecal suspension. Next, the supernatant was removed after the fecal suspension was centrifuged with centrifuge (5804R, Eppendorf, Hamburg, Germany) at 6,000 × *g* for 15 min. We resuspended the precipitate in sterile saline containing 10% glycerol, and finally stored at −80 °C for subsequent FMT experiment. Before FMT, the frozen fecal suspension was thawed in a water bath at 37 °C. Upon frozen fecal suspension thawing, it was diluted with sterile saline to 1 × 10^9^ colony forming units (CFU) per milliliter and fecal suspension was gavaged to receiver hens as soon as possible at room temperature.

##### Experimental design

Fourteen 24-week-old Hy-Line gray commercial laying hens were selected and fed HFD for 16 weeks to induce FLHS after an adaptation period of one week, and then were randomly divided into two groups, with 7 replicates per group and one hen per replicate. The hens were raised in ladder cages with one bird per cage and continued to receive HFD. All the hens were treated for 7 d with a antibiotics mixture of neomycin (1 g/L; N6386, Sigma-Aldrich, Saint Louis, USA), ampicillin (1 g/L; A5354, Sigma-Aldrich, Saint Louis, USA), metronidazole (1 g/L; M3761, Sigma-Aldrich, Saint Louis, USA) and vancomycin (0.5 g/L; SBR00001, Sigma-Aldrich, Saint Louis, USA) in their drinking water [38], and then a 4-d washout period ensured elimination of the residual antibiotics as previously described [39]. Then, hens of HFD_GBE-FMT group (*n* = 7) were administered 5 mL of fecal bacteria suspension (1 × 10^9^ CFU/mL), and hens of CON group (*n* = 7) were given an equivalent volume of sterile saline by oral gavage each day at 2-week intervals until 8 weeks.

The day before the end of the experiment, all laying hens were fasted for 8 h, but water was offered ad libitum. Then all laying hens were weighed, and blood samples were collected from the wing vein. The blood was centrifuged at 3,000 × *g* for 10 min at 4 °C and the serum was collected and stored at −80 °C. At the end of the experiment, all laying hens were euthanized via cutting the jugular vein. The liver was imaged and weighed, and approximately 1 cm of liver tissue was taken and fixed in 4% paraformaldehyde for histopathological evaluation. Three small pieces of liver tissue were taken, divided them into three centrifuge tubes, snap-frozen in liquid nitrogen and stored at −80 °C. One tube is used to measure the fat content of the liver, one tube is used to measure the antioxidant activity of the liver, and the other tube is used for mRNA analysis. The heart and abdominal fat were weighed.

### Serum biochemistry analysis

Levels of serum glucose (GLU), triglycerides (TG), total cholesterol (TC, T-CHO), alanine transaminase (ALT), aspartate transaminase (AST) and alkaline phosphatase (ALP) were measured by the automatic biochemical analyzer (KHB ZY-1280, Shanghai Kehua Bio-engineering Co., Ltd., China) as instructed by the manufacturer.

### Measurement of hepatic lipid contents

Hepatic lipids were extracted according to Folch method [40] with slight modifications. Briefly, 50 mg of liver tissue was weighted and homogenized in 1 mL of chloroform:methanol (2:1) before agitation at 50 Hz for 30 s with refrigerated high-throughput tissue grinder (SCIENTZ-48L, Ningbo SCIENTZ Biotechnology Co., Ltd., China). 200 µL of distilled water was added and vortex to mix, following by centrifugation at 4,000 × *g* for 10 min and the lower solvent layer was extracted and then dried with a centrifugal vacuum concentrator (CV200, Beijing JM Technology Co., Ltd., China). The residue was resuspended with an equivalent volume of PBS supplemented with 5% Triton X-100 as a total lipid extract sample. The contents of TG and TC in liver were assayed by using commercial kits based on manufacturer’s guidelines (Biosino Biotechnolgy and Science Inc., Beijing, China).

### Histopathological evaluation

Fresh samples of liver were resected and fixed with 4% paraformaldehyde at room temperature. The samples were dehydrated and embedded in paraffin, and then sectioned and further stained with hematoxylin–eosin staining (HE). Images were obtained using a LIOO 3.7 for digital camera software (Leica DM750, Leica Biosystems, Nussloch, Germany). The non-alcoholic fatty liver disease (NAFLD) activity score (NAS) of pathological sections of liver tissues, a semi-quantitative evaluation, was conducted based on the levels of hepatocyte steatosis, inflammation within the lobules and ballooning degeneration of hepatocyte [41].

### Measurement of serum and liver antioxidant indexes

0.2 g of liver tissue was weighted and homogenized in 2 mL of physiological saline, following by centrifugation at 3,500 × *g* for 15 min and the lower solvent layer was obtained as liver homogenate. Then, the BCA protein concentration assay kit (P0012 BCA, Beyotime Biotechnology, Nantong, Jiangsu, China) was used to detect the protein content in liver homogenate. The contents of malondialdehyde (MDA), superoxide dismutase (T-SOD), total antioxidant capacity (T-AOC), GSH and glutathione peroxidase (GSH-Px) in serum and liver homogenate were determined with microplate reader (Epoch 2, BioTek Instruments, Inc., Winooski, Vermont, USA) by using commercial kits based on manufacturer’s guidelines (Nanjing Jiancheng Bioengineering Institute, Jiangsu, China).

### Total RNA isolation and real-time PCR

Total RNA from tissues or cells were isolated using the TRIzol method, and then used to synthesize the first-strand cDNA with the BeyoRT™ II cDNA Synthesis Kit with gDNA Eraser (D7170M, Beyotime Biotechnology, Nantong, Jiangsu, China). Real-time quantitative polymerase chain reaction (RT-qPCR) was carried out with BeyoFast™ SYBR Green qPCR Mix (2X, Low ROX) (D7262, Beyotime Biotechnology, Nantong, Jiangsu, China) on an Applied Biosystems® QuantStudio™ 7 Flex Real-Time PCR System (QuantStudio7 Flex, Thermo Fisher Scientific, Waltham, USA). The results were analyzed using the 2^−ΔΔCt^ method with β-actin serving as the reference. The primers used were synthesized by Sangon Biotech (Shanghai, China), and listed in Table 2.

**Table 2:** The primers for RT-qPCR assays

Target genes	Forward primer (5′→3)	Reverse primer (5′→3′)
*β-actin*	GAGAAATTGTGCGTGACATCA	CCTGAACCTCTCATTGCCA
*SREBP-1c*	CCCGAGGGAGACCATCTACA	GGTACTCCAACGCATCCGAA
*FAS*	CCAACGATTACCCGTCTCAA	CAGGCTCTGTATGCTGTCCAA
*SCD1*	CCAGCGGAGATACTACAAGCC	CCGATTGCCAAACATGTGAGC
*GPAT1*	TCCATCGAGACCTAATGATAC	TAGACATCACAGCACAGGAC
*ChREBP*	GATGAGCACCGCAAACCAGAGG	TCGGAGCCGCTTCTTGTAGTAGG
*HMGCR*	CATAGGTGGCTACAACG	TACGCTCCATCAAAGTG
*LXRα*	CAAAGGGAATGAATGAGC	AGCCGAAGGGCAAACAC
*PPARγ*	GCAGGAACAGAACAAAGAAG	TGCCAGGTCACTGTCATCTA
*PPARα*	TGTGGAGATCGTCCTGGTCT	CGTCAGGATGGTTGGTTTGC
*FXR*	AGTAGAAGCCATGTTCCTCCGTT	GCAGTGCATATTCCTCCTGTGTC
*ABCA1*	TCAATCACCCGCTCAACT	CTGGCAGGAACAAAGGAC
*ACOX1*	ATGTCACGTTCACCCCATCC	AGGTAGGAGACCATGCCAGT
*CPT1*	TCGTCTTGCCATGACTGGTG	GCTGTGGTGTCTGACTCGTT
*L-FABP*	GAAGAGTGTGAGATGGAGCTGCTG	GGTGATGGTGTCTCCGTTGAGTTC
*TNF-α*	GGATGGATGGAGGTGAAAGTAG	TGATCCTGAAGAGGAGAGAGAA
*IL-6*	CGCCCAGAAATCCCTCCTC	AGGCACTGAAACTCCTGGTC
*TLR4*	CCACTATTCGGTTGGTGGAC3	ACAGCTTCTCAGCAGGCAAT
*NF-κB*	CTCTCCCAGCCCATCTATGA	CCTCAGCCCAGAAACGAAC

### 16S rRNA gene sequencing and analysis

Bacterial genomic DNA was extracted from cecal content or ileal content of animals with a QIAamp Fast DNA stool Mini Kit (51604, Qiagen, Dusseldorf, North Rhine-Westphalia, Germany) for subsequent 16S rRNA gene sequencing. Qualified DNA samples were purified and amplified using specific bacterial primers targeting the variable region V3–V4 of the 16S rRNA gene, and amplification primers were 341F (5′-CCTACGGGNBGCASCAG-3′) and 805R (5′-GACTACNVGGGTATCTAATCC-3′) [42]. Purified amplicons were pooled in equimolar and then paired-end sequencing was performed on an Illumina Hiseq 2500 sequencing platform at the Institute of Microbiology, Chinese Academy of Sciences (Beijing, China) according to standard protocols. The generated raw fastq files were quality-filtered and taxonomically analyzed using QIIME2 (V2019.7) [43]. In brief, primers of imported sequences were removed via Cutadapt (V2.4) [44], and then DADA2 was used to filter and denoise sequences, remove chimeras, infer amplicon sequence variants (ASVs) and generate the abundance table [45]. The ASVs were clustered with 100% similarity, and species annotation was performed for each ASV using the Naive Bayes classifier method based on the SILVA database [46]. Alpha diversity of each sample was assessed using the vegan package for R to assess bacterial richness and evenness. The weighted Unifrac based principal co-ordinates (PCoA) analysis was conducted to reflect differences in community structure of gut microbiota in each group, ANOSIM analysis was used to evaluate the statistical differences among groups.

### GC–MS analysis of SCFAs

Targeted metabolomics was performed with GC–MS to quantify the concentrations of SCFAs in cecal content. Briefly, for cecal content samples, the extraction procedures have been carried out 4 °C to protect the volatile SCFAs. 50 mg cecal content were diluted with distilled water (4 × the volume), tubes were homogenized and centrifuged at 12,000 × *g* for 15 min at 4 °C. Following centrifugation supernatants were transferred into centrifuge tubes for the derivatization reaction. 200 µL of 25% metaphosphoric acid solution was added to the supernatants, the solution was kept standing for 0.5 h with an environmental temperature of 4 °C, and then centrifuged at 12,000 × *g* for 10 min at 4 °C. Next, transfered the obtained supernatant to glass bottles and sent to the Institute of Animal Sciences of Chinese Academy of Agricultural Sciences at a low temperature for SCFAs determination using GC-MS (Agilent 5975C, Santa Clara, CA, USA). The concentration gradient of standard curve was 0 to 100 μg/mL for low concentration and 0 to 600 μg/mL for high concentration. SCFAs levels in the cecal content were normalized to the weight.

### Statistical analysis

Statistical analysis was performed using GraphPad Prism version 8.0 (GraphPad Software, San Diego, CA, USA) and data are shown as means ± SEM. Significant differences between the two groups were assessed by unpaired two-tailed Student’s *t*-test or Mann–Whitney U test for samples that were not normally distributed. Data sets involving more than two groups were evaluated by one-way analysis of variance (ANOVA) followed by the Least Significant Difference (LSD) or by the non-parametric Kruskal–Wallis test with IBM SPSS Statistics 24.0 (SPSS Inc., Chicago, IL, USA). *P* < 0.05 was considered to be statistically significant.

## Results

### GBE supplementation ameliorates the HFD-induced FLHS in laying hens

**Fig. 1:**
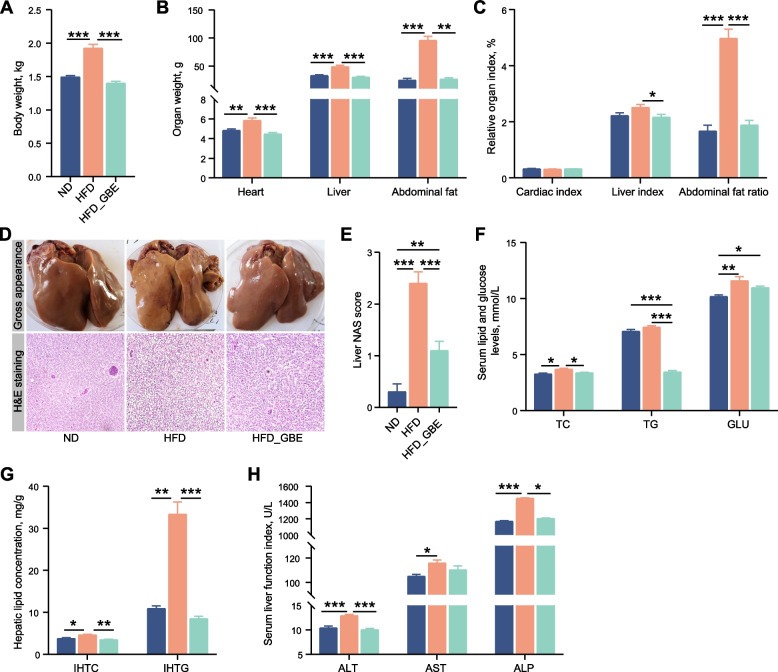
GBE relieves HFD-induced FLHS in laying hens. **A** Body weight. **B** Organ weight. **C** Organ index (%, organ weight (g)/body weight (g) × 100%). **D** Representative photomicrographs of liver tissues with H&E staining and phenotype pictures. **E** Assessment of non-alcoholic fatty liver disease (NAFLD) activity score (NAS) of liver tissues based on histological sections. **F** Serum lipid and glucose levels. **G** Hepatic lipid levels. **H** Serum liver function index. Data are presented as the mean ± SEM; *n* = 10 hens per group. Statistical analysis was performed using one-way ANOVA followed by the LSD. ^*^P < 0.05, ^**^P < 0.01, ^***^P < 0.001

To evaluate the therapeutic effects of GBE on FLHS, we treated HFD-induced laying hens with 0.12% GBE for a period of 16 weeks. The body weight, heart weight, liver weight, abdominal fat weight and abdominal fat ratio of the laying hens in the HFD group were significantly elevated compared with the ND group, but GBE treatment effectively decreased these indexes compared with the HFD group (Fig. 1A–C). According to liver tissue H&E staining and phenotype pictures, FLHS laying hens exhibited severe hepatic steatosis with yellowish and larger liver, a great number of fat vacuoles and higher liver NAS score, which was alleviated after GBE treatment (Fig. 1D–E). Additionally, FLHS laying hens showed a significant increase in the concentrations of TC and GLU in the serum and intrahepatic lipid contents (IHTG and IHTC). In contrast, GBE intervention obviously masked off the effects of the HFD on these disturbed metabolic parameters in FLHS laying hens, implying its regulative effects on hepatic lipid metabolism (Fig. 1F–G). ALT, AST and ALP activities in serum are often used as reliable indicators for assessing liver damage. When the hepatocytes are damaged, these enzymes enter the circulatory system from the hepatocytes, thereby increasing the enzyme activities in the serum [47]. Notably, these enzymes were significantly increased in HFD group compared with the ND group, while the HFD_GBE group showed significant improvement after GBE intervention (Fig. 1H). Overall, the above results indicate the therapeutical effects of GBE on the pathological progression of FLHS.

### Effects of GBE on antioxidant activity of FLHS laying hens

**Fig. 2:**
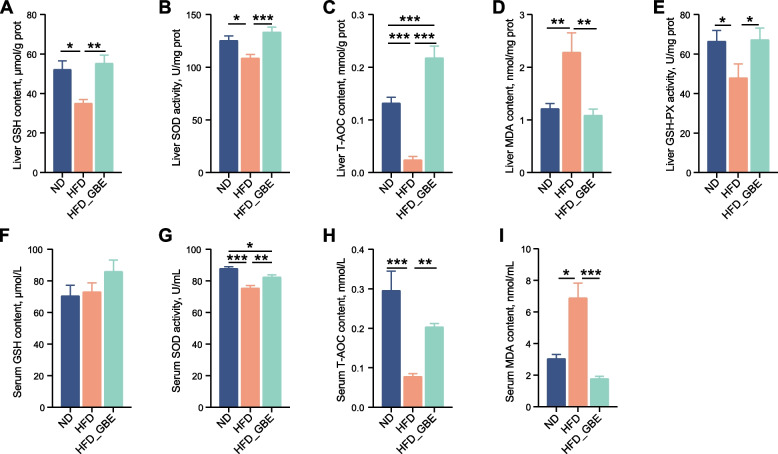
Effect of GBE on antioxidant indices in liver and serum. **A** The reduced GSH content of liver tissues. **B** The SOD activity in liver tissues. **C** The T-AOC of liver tissues. **D** The MDA content of liver tissues. **E** The GSH-PX activity in liver tissues. **F** The reduced GSH content of serum. **G** The SOD activity in serum. **H** The T-AOC of serum. **I** The MDA content of serum. Data are presented as the mean ± SEM; *n* = 10 hens per group. Statistical analysis was performed using one-way ANOVA followed by the LSD. ^*^P < 0.05, ^**^P < 0.01, ^***^P < 0.001

Studies have pointed out that GBE exerts protective and antioxidative effects by up-regulating the expression of SOD and GSH which in turn inhibits the synthesis of ROS [48, 49], we thereby measured the antioxidant activity in liver and serum of laying hens. Compared with the ND group, the hepatic GSH, SOD, T-AOC and GSH-PX activities of laying hens in HFD group were decreased, and the hepatic MDA contents were significantly increased. Whereas GBE treatment significantly enhanced the activities of GSH, SOD, T-AOC and GSH-PX, and decreased the activity of MDA. Interestingly, similar results were also found in serum (Fig. 2A–E). GBE caused increase in the level of SOD and T-AOC, and reduction of 74.2% in the level of MDA (Fig. 2F–I). Thus, GBE improves the antioxidant capacity of HFD-induced FLHS laying hens.

### GBE decreased gene expression of lipid synthesis and inflammatory cytokines in liver of FLHS laying hens

**Fig. 3:**
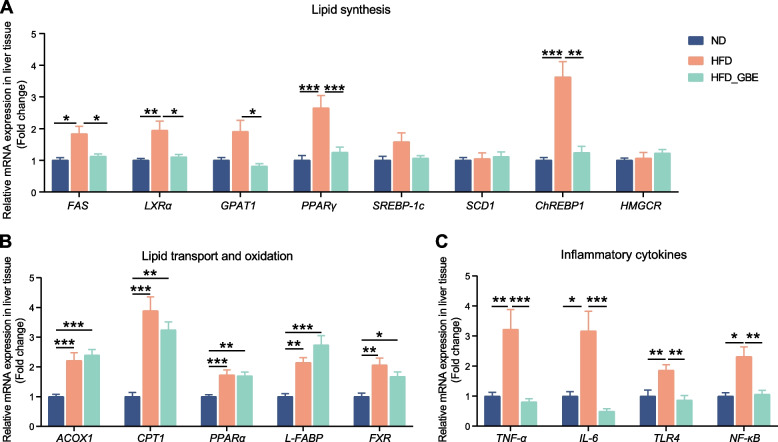
Effects of GBE supplementation on the expression of genes related to lipid metabolism and inflammatory factors in the liver of laying hens. The relative mRNA expression of lipid synthesis (**A**), lipid transport and oxidation (**B**) and inflammatory cytokines (**C**) related genes in liver tissues. Data are presented as the mean ± SEM; *n* = 10 hens per group. Statistical analysis was performed using one-way ANOVA followed by the LSD or non-parametric Kruskal–Wallis test. ^*^P < 0 .05, ^**^P < 0.01, ^***^P < 0.001

To further reveal the mechanism of GBE in alleviating FLHS, we measured the related genes expression of de novo lipogenesis, the lipid beta-oxidation, and the inflammatory cytokine from the liver in the FLHS laying hens with or without GBE treatment. Compared with the ND group, the HFD group significantly increased the expression levels of genes related to lipid synthesis, such as fatty acid synthase (*FAS*), liver X receptor alpha (*LXRα)*, peroxisome proliferator-activated receptor gamma (*PPARγ*) and carbohydrate response element-binding protein 1 (*ChREBP1*) (Fig. 3A). The intake of HFD also significantly up-regulated the expression level of genes related to lipid transport and oxidation, such as acyl-CoA oxidase 1 (*ACOX1),* peroxisome proliferator-activated receptor alpha (*PPARα*), liver fatty acid binding protein (*L-FABP*) and farnesoid X receptor (*FXR*) (Fig. 3B). In addition to the excessive accumulation of lipids in livers, steatohepatitis is also an important pathological feature of FLHS. HFD increased the expression levels of genes related to inflammatory cytokine, such as tumor necrosis factor alpha (*TNF-α*), interleukin 6 (*IL-6*), toll-like receptors 4 (*TLR4*) and nuclear factor kappa-B (*NF-κB*) (Fig. 3B). After GBE intervention, the treated group displayed markedly reduced expression levels of genes related to lipid synthesis, such as *FAS*, *LXRα*, glycerol-3-phosphate acyltransferase1 (*GPAT1*), *PPARγ* and *ChREBP1* (Fig. 3A)*,* and also significantly downregulated expression levels of several inflammatory cytokine markers (*TNF-α, IL-6, TLR4* and *NF-κB*) (Fig. 3C), despite no effect on the expression of lipid transport and oxidation related genes compared with the HFD group (Fig. 3B). These data support the effectiveness of GBE in attenuating FLHS by reducing the expression of genes related to lipid synthesis and inflammatory cytokines in the liver.

### The anti-FLHS effect of GBE is associated with gut microbiota changes

**Fig. 4:**
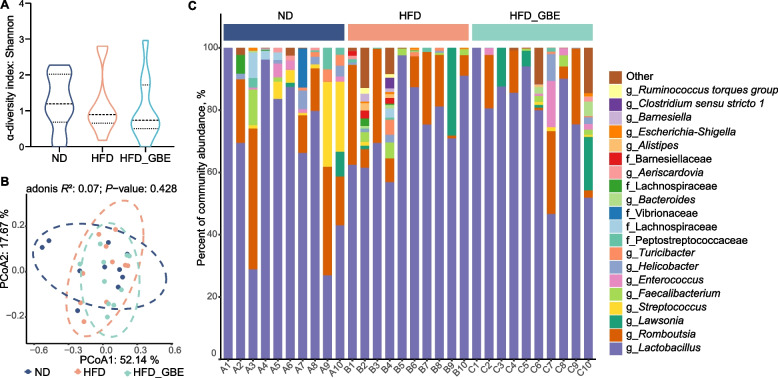
GBE does not alter ileal microbial diversity and composition of FHLS laying hens. **A** Alpha diversity of ileal microbiota in different groups. Data were analyzed by nonparametric Kruskal–Wallis test. **B** Principal coordinate analysis (PCoA) of ileal microbiota in different groups. PCoA plots were generated using OUT abundance data according to the Bray–Curtis distance, and statistical significance was measured using adonis analysis. **C** Relative abundance of top 20 genera in each sample.* n* = 10 hens per group

**Fig. 5:**
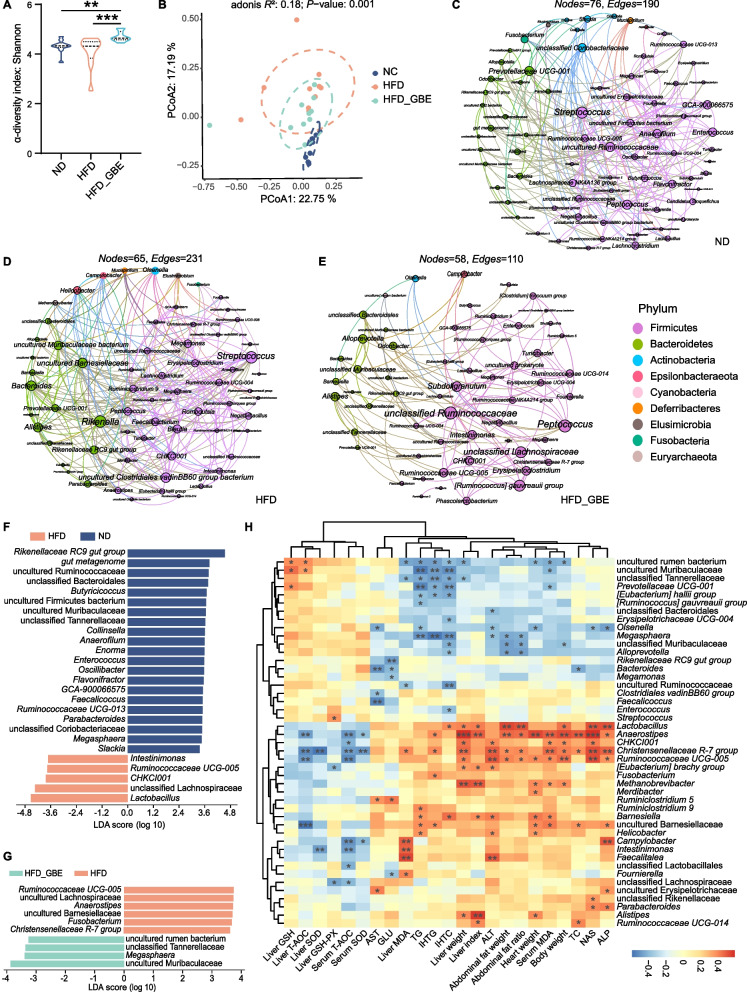
The impacts of GBE on cecal microbiota of HFD-induced FLHS laying hens. **A** The alpha diversity of cecal microbiota in different groups. Data were analyzed by nonparametric Kruskal–Wallis test. **B** Principal coordinate analysis (PCoA) of cecal microbiota in different groups. PCoA plots were generated using OUT abundance data according to the Bray–Curtis distance, and statistical significance was measured using adonis analysis. **C–E** Gut microbial co-occurrence network analysis based on core genus (average relative abundance > 0.1% in the ND, HFD and HFD_GBE groups). Different colors of nodes indicate different phylum levels. Edges represent significant correlations (*P* < 0.05). **F**–**G** Differences in the bacterial communities between different groups at the genus level. Significant differences in Linear discriminant analysis (LDA) scores (*P* < 0.05) were produced between groups (Wilcoxon’s test). The threshold of the logarithmic LDA score was 2.0. **H** Spearman’s correlation analysis between related indicators of metabolic disorders and cecal microbes at the genus level: ^*^P < 0.05, ^**^P < 0.01, ^***^P < 0.001. Red represents positive correlations and blue represents negative correlations. *n* = 10 hens per group

To assess the effect of GBE on the gut microbiota of FLHS laying hens, we performed 16S rRNA gene sequencing on the cecal and ileal contents of laying hens. We found that GBE had no significant influence on α-diversity (as analysed by the Shannon index) and β-diversity (as determined by PCoA analysis) of ileal microbiota (Fig. 4A and B), and no differentially abundant taxa were observed at the genus levels (Fig. 4C). In contrast, both the Shannon analysis and principal coordinate analysis (PCoA) showed significant differences of the cecal microbiota among the three groups (Fig. 5A and B). The co-occurrence network analysis based on the relative abundance of genus showed that there were 65 nodes and 231 edges in the HFD group, while ND group had 76 nodes and 190 edges, indicating that bacterial network of the HFD group is more complex than that of the ND group. Moreover. The core genera of the ND group and the HFD group are contained in 7 phyla and 8 phyla, respectively. Notably, HFD_GBE group contains only 58 nodes, 110 edges and 5 phyla, indicating GBE intervention reversed the complex bacterial network caused by HFD (Fig. 5C–E). Subsequent linear discriminant analysis (LDA) effect size (LEfSe) results revealed obvious differences in the cecal bacterial communities between different groups. At the genus level, compared with the ND group, HFD reduced several beneficial bacteria, such as *Butyricicoccus*, *Enterococcus*, *Flavonifractor*, *Faecalicoccus*, *Parabacteroides* and *Megasphaera*, but enhanced *Intestinimonas*, *Ruminococcaceae UCG-005*, *CHKCI001*, unclassified Lachnospiraceae and *Lactobacillus* (Fig. 5F). After GBE intervention, the abundances of uncultured rumen bacterium, unclassified *Tannerellaceae, Megasphaera,* and uncultured Muribaculaceae were significantly enriched, but the abundances of *Ruminococcaceae UCG-005*, *Anaerostipes*, *Fusobacterium* and *Christensenellaceae R-7 group*, etc. were significantly reduced (Fig. 5G). Further correlation analysis found that GBE-enriched bacteria had a significant negative correlation with the markers of FLHS and a significant positive correlation with antioxidant capacity, while HFD-enriched bacteria showed the opposite results (Fig. 5H). Notably, *Megasphaera* was the only genus that was significantly reduced by HFD but rescued by GBE (Fig. 5F and G), which was significantly negatively correlated with different FLHS markers including abdominal fat weight and ratio, ALT, IHTG, IHTC and serum TG (Fig. 5H). Collectively, these data suggested that GBE may be involved in the alleviation of FLHS through modulating cecal microbiota rather than ileal microbiota, especially the abundance of *Megasphaera* in the cecum.

### GBE increases the cecal SCFAs concentration

**Fig. 6:**
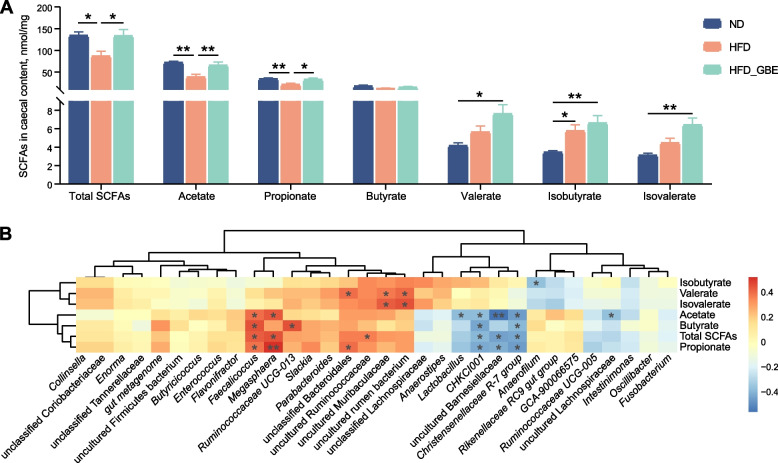
Targeted metabolomic analyses of SCFAs levels using GC–MS in laying hens. **A** SCFAs concentration in cecal contents of laying hens. **B** Correlation analysis of SCFAs concentration and the differentially abundant bacteria. Red represents positive correlations and blue represents negative correlations. ^*^P < 0.05, ^**^P < 0.01, ^***^P < 0.001. *n* = 10 hens per group

The microbiota-derived SCFAs play an important role in alleviating MAFLD [17]. We observed that the HFD feeding led to a total reduction in SCFAs levels of cecal contents, especially decreased the concentrations of acetate and propionate (Fig. 6A). However, GBE treatment remarkably elevated the total SCFAs, acetate and propionate levels compared with the HFD group. Correlation analysis between the SCFAs concentration and the differentially abundant microbes revealed that uncultured rumen bacterium, *Megasphaera* and uncultured Muribaculaceae enriched in the HFD_GBE group and *Faecalicoccus*, *Ruminococcaceae UCG-013*, unclassified Bacteroidales and uncultured Ruminococcaceae enriched in the ND group were significantly positively correlated with the SCFAs concentration (Fig. 6B). In contrast, *Lactobacillus*, *CHKCI001*, uncultured Barnesiellaceae, *Christensenellaceae R-7 group* and uncultured Lachnospiraceae enriched in the HFD group were significantly negatively correlated with the SCFAs concentration (Fig. 6B). These results demonstrated the ability of GBE to promote the growth of SCFAs-producing bacteria.

### FMT attenuates HFD-induced FLHS in laying hens

**Fig. 7:**
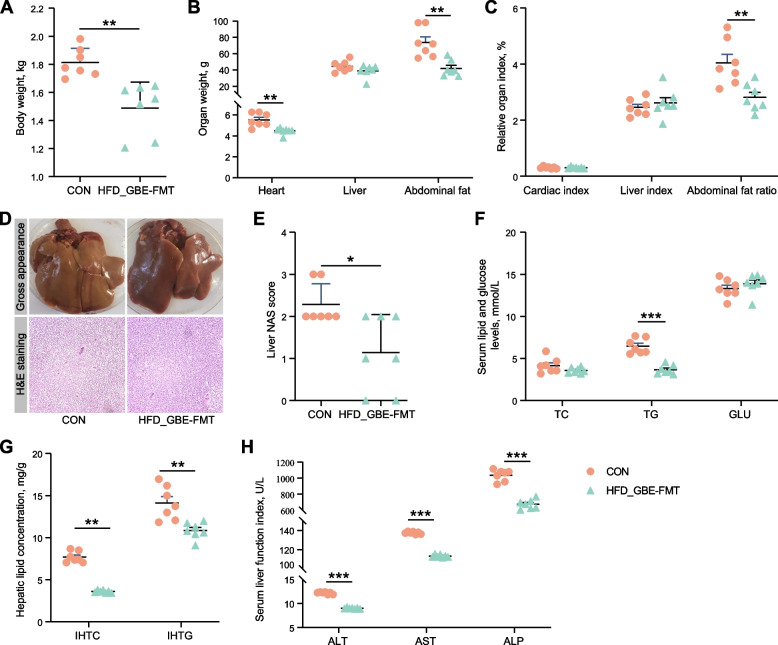
FMT attenuates HFD-induced FLHS in laying hens. **A** Body weight. **B** Organ weight. **C** Organ index. **D** Representative photomicrographs of liver tissues with H&E staining and phenotype pictures. **E** Assessment of non-alcoholic fatty liver disease (NAFLD) activity score (NAS) of liver tissues based on histological sections. **F** Serum lipid and glucose levels. **G** Hepatic lipid levels. **H** Serum liver function index. Data are presented as the mean ± SEM; *n* = 7 hens per group. Statistical analysis was performed using independent-samples *t* test. ^*^P < 0 .05, ^**^P < 0.01, ^***^P < 0.001

To investigate whether GBE-altered gut microbiota had therapeutic effects on FLHS, we performed a FMT experiment. The results showed that HFD-induced FLHS laying hens orally treated with fecal bacterial suspension from GBE-treated donors significantly decreased the body weight, heart weight, abdominal fat weight and abdominal fat ratio (Fig. 7A–C). Notably, FMT showed apparent decrease in size and conspicuous color change toward to fuchsia from yellowish relative to the model group, and effectively reduced the fat vacuoles number in liver of FLHS laying hens (Fig. 7D). The liver NAS score in the FMT group was significantly lower than the model group (Fig. 7E). Additionally, FMT exhibited little improvements in serum TC and GLU, but significantly decreased lipid deposition (as indicated by serum TG, IHTC and IHTG) and liver injury (Fig. 7F–H). These findings further support the notion that GBE alleviates FLHS in laying hens is mediated by gut microbes.

### Effect of FMT on antioxidant activity in liver and serum of FLHS laying hens

**Fig. 8:**
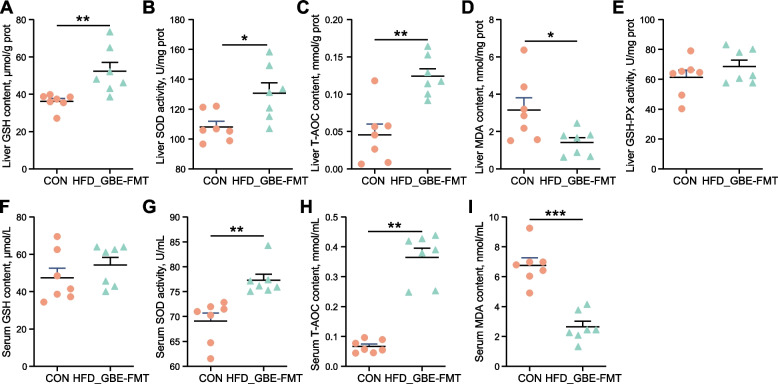
Effect of FMT on antioxidant activity in liver and serum. **A** The reduced GSH content of liver tissues. **B** The SOD activity in liver tissues. **C** The T-AOC of liver tissues. **D** The MDA content of liver tissues. **E** The GSH-PX activity in liver tissues. **F** The reduced GSH content of serum. **G** The SOD activity in serum. **H** The T-AOC of serum. **I** The MDA content of serum. Data are presented as the mean ± SEM; *n* = 7 hens per group. Statistical analysis was performed using independent-samples *t* test. ^*^P < 0 .05, ^**^P < 0.01, ^***^P < 0.001

FMT significantly decreased the activities of MDA in liver of FLHS laying hens, and the hepatic GSH, SOD and T-AOC activities were significantly increased by 44.83%, 20.95%, and 172.55% compared with those in the model group, respectively (Fig. 8A–E). Likewise, FMT significantly reversed the effects of HFD on serum SOD, T-AOC and MDA activities (Fig. 8F–I). Overall, FMT enhances the antioxidant capacity of FLHS laying hens.

### Effect of FMT on gene expression of lipid metabolism and inflammatory factors in liver of FLHS laying hens

**Fig. 9:**
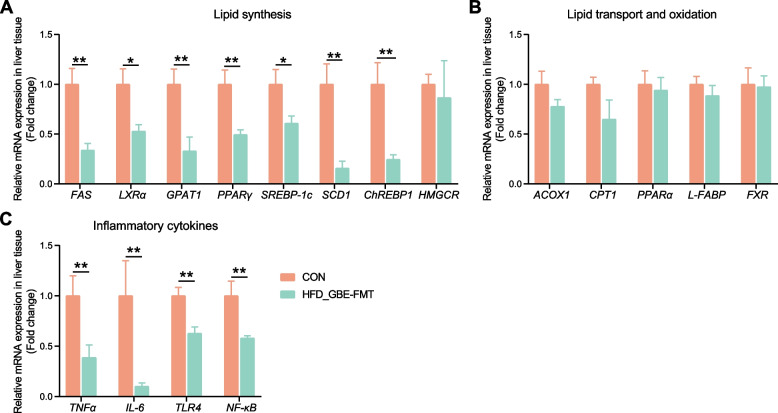
Effects of FMT on the expression of genes related to lipid metabolism and inflammatory factors in the liver of FLHS laying hens. The relative mRNA expression of lipid synthesis (**A**), lipid transport and oxidation (**B**) and inflammatory cytokines (**C**) related genes in liver tissues. Data are presented as the mean ± SEM; *n* = 7 hens per group. Statistical analysis was performed using independent-samples *t* test or non-parametric Mann-Whitney U test. ^*^P < 0 .05, ^**^P < 0.01, ^***^P < 0.001

qPCR analysis of the liver of FLHS laying hens revealed that FMT significantly decreased the expression levels of genes related to lipid synthesis, such as *FAS*, *LXRα*, *GPAT1*, *PPARγ*, *SREBP-1c*, *SCD1* and *ChREBP1* (Fig. 9A), and increased the expression levels of genes related to inflammatory cytokine, such as *TNF-α, IL-6, TLR4* and *NF-κB* (Fig. 9C); however, no effect was observed on the expression levels of genes related to lipid transport and oxidation (Fig. 9B). Altogether, FMT inhibited lipid synthesis and inflammatory responses of FLHS laying hens.

## Discussion

As a natural antioxidant, GBE has been reported to be beneficial to health by helping the body resist harmful oxidative stress and reducing the infiltration of inflammatory cells and inflammatory cytokines [20, 49]. Currently, GBE is widely used in the alleviation of metabolic diseases such as obesity and MAFLD due to its excellent lipid-lowering effect [27–29], while the pharmacological mechanism of GBE on anti-FLHS remains unelucidated. In the current study, we found that the anti-FLHS effect of GBE was associated with the reshaping of the chicken gut microbiota.

Our study showed that FLHS laying hens with GBE intervention eliminated hepatic steatosis, inflammation and liver injury, and increased antioxidant activity. These therapeutic effects largely meet the recommended criteria for the treatment of MAFLD [50]. Interestingly, the anti-FLHS function of GBE in FLHS laying hens was transferred through FMT to new receivers, which further demonstrated that the anti-FLHS effects of GBE was associated with its modulation on gut microbiota. Our study provided new clues in explaining the metabolic benefits of GBE.

In this study, the disorder of lipid metabolism in FLHS model was effectively reversed by supplementing GBE, which was consistent with the fact that GBE could inhibit lipid accumulation in the liver of obese rats reported previously [28, 51]. *FAS*, *SREBP-1c*, *GPAT*, *PPARγ, LXRα*, *SCD1* and *ChREBP1* are key genes involved in de novo lipid synthesis. *FAS* is a key enzyme that catalyzes fatty acid synthesis [52]. *SREBPs* is an important transcription factor that regulates the biosynthesis and uptake of cholesterol and fatty acids [53]. *GPAT1* is the rate-limiting enzyme for the synthesis of glycerophospholipids and triglycerides [54]. *PPARγ* mainly promotes lipogenesis and preadipocyte differentiation [55] and its upregulation contributes to hepatic steatosis [56]. *LXRα*, as a ligand-dependent nuclear receptor, which can form a positive feedback loop with *SREBP-1c* and enhance lipogenesis in the liver and directly activate target genes *ACC*, *FAS* and *SCD1* by the binding of LXREs to their promoter regions [2, 57]. In the present study, GBE inhibited lipid synthesis by significantly downregulated the gene expression of hepatic *FAS*, *LXRα*, *GPAT1*, *PPARγ* and *ChREBP1,* thus alleviating the fat accumulation in FLHS laying hens.

Fatty acid accumulation is not only a characteristic of FLHS, but also causes insulin resistance, lipid peroxidation, hepatic damage, inflammatory response, and energy metabolism disorders [58]. A study found that excessive deposition of palmitic acid in the liver can activate *NF-κB* and cause an inflammatory response [59]. *TLR4* is expressed in hepatic stellate cells (HSCs), where it triggers an inflammation-like response with *NF-κB* activation and further *NF-κB*-dependent gene expression [60]. *TNF-α* is the most prominent “first-line” cytokines [61], which stimulates and induces ROS production and lipid peroxidation, and it also activates oxidative stress response genes which amplify and prolong inflammation [62]. Meanwhile, Oxygen free radicals and *TNF-α* released during inflammation could also activate and induce *NF-κB*, and then upregulate the expression of *TNF-α*, involved in immune and inflammatory responses [57, 63]. Of note, *TNF-α* can induce the release of *IL-6* from Kupffer cells in the liver, which hinders the transport and secretion of TG [64]. On the other hand, elevated *TNF-α* can lead to hepatic TG accumulation and steatosis, which in turn activates *NF-κB*, thus form a vicious cycle of aggravating liver damage [65]. Our results showed that GBE supplementation significantly reduced the activity of serum ALT, AST and ALP in the FLHS laying hens, reflecting alleviation of liver damage. Likewise, the expression levels of *TLR4*, *NF-κB*, *TNF-α* and *IL-6* were downregulated, which is consistent with previous studies that GBE ameliorates inflammation by decreasing the expression of *NF-κB*p65, *TNF-α* and IL-6 [63, 66]. Our results together with previous findings suggested that GBE could inhibit the activation of *TNF-α*, *IL-6* and *TLR4*-*NF-κB* pathway, thereby attenuating inflammation cascade effects and tissue damage.

Oxidative stress induces hepatic lipid metabolism disturbance, which is also an important mechanism for liver-related diseases [67]. It has been shown that oxidative stress and its consequent lipid peroxidation aggravate the free radicals chain reaction and activate inflammatory mediators [63]. MDA activities are often used as a marker of free radicals-induced lipid peroxidation and as an indicator of oxidative damage [63], which can interact with *NF-κB* to increase the release of *TNF-α* [68]. As a primary defense, SOD reduces the activation of inflammatory mediators and oxidative stress [63]. It has been reported that SOD treatment significantly reduces lipid oxidation and improves colonic inflammation in ulcerative colitis [69]. Of note, GBE acts as a free radical scavenger with SOD-like activity [63], which reduces lipid peroxidation in CCl_4_-induced liver fibrosis by enhancing SOD activities [70]. GSH, T-AOC and GSH-PX are other commonly used indicators to evaluate the degree of oxidative stress. In the present study, GBE treatment significantly increased the activities of SOD and T-AOC, and reduced MDA contents in the liver and serum, indicating that GBE increased the body’s total antioxidant capacity and effectively inhibited lipid peroxidation induced by HFD.

Gut microbiota and its metabolites play an important role in host metabolism. Our results showed that GBE supplementation dramatically reversed the HFD-induced flora imbalance of FLHS laying hens, and transplanting GBE-altered intestinal flora to FLHS laying hens improved hepatic steatosis, inflammation and anti-oxidation. This result strongly suggested that the anti-FLHS effect of GBE was related to its modulation of gut microbiota. Interestingly, *Megasphaera* is the only genus that was significantly decreased by HFD but enriched by GBE, and was significantly negatively correlated with FLHS traits. *Megasphaera* is obligately anaerobic, Gram-negative, non-motile, coccoid bacteria, which belongs to the family Veillonellaceae under the order Veillonellales, class Negativicutes, and phylum Firmicutes [71]. Studies have found that *Megasphaera* is a dominant genus of gut microbiota in different life stages of primates, and is also a key driving factor for the long-term development of gut microbiota in adults [72]. *Megasphaera* isolated from the human gut have been found to contain a broad range of carbohydrate active enzymes, and oligopeptide transport systems that enables them to use carbohydrates, as well as a broad range of amino acids as carbon sources [73]. These capabilities endow *Megasphaera* with a competitive advantage in the gut microenvironment especially when the microenvironment changes. Previous studies have demonstrated that *Megasphaera* is able to produce SCFAs, including acetate, propionate, butyrate, valerate, caproate, isobutyrate, isovalerate and isocaproate [71, 74]. In particular, *Megasphaera* can metabolize lactate and buffer fat to produce acetate, propionate and butyrate [75, 76]. These SCFAs may regulate lipid levels by downregulating cholesterol biosynthesis and increasing bile acid excretion [77–79]. A recent study showed that dietary *Lactobacillus delbrueckii* lower serum TG levels of growing-finishing pigs via enhancing the abundance of bacteria such as *Megasphaera* and butyrate content in the colon [80]. Another study showed that *Megasphaera* exerts an important role in lowering blood lipids in hyperlipidemia animals [76]. Compared with HFD-induced monkeys, the abundance of *Megasphaera* was found dramatically increased in high-fat-diet-tolerant cynomolgus monkeys (HFD-T). Notably, the abundance of *Megasphaera* was also dramatically increased in HFD-induced rats after transplantation of fecal microbiota from HFD-T monkeys and this increasing trend was consistent with that in monkeys. All these facts indicate that *Megasphaera* may have beneficial effects on lipid metabolism-related diseases. Therefore, we speculate that the *Megasphaera* might play a critical role in the treatment of FLHS, which deserved to be further investigated.

## Conclusions

In conclusion, we demonstrated that GBE is effective in improving steatosis, antioxidant activity, inflammation and FLHS in laying hen model. We also evidenced that these effects are probably mediated by GBE-altered gut microbiota. Our work provides fundamental evidence for the promising therapeutic potential of GBE in the treatment of FLHS and related metabolic diseases, and also shed lights on manipulating the chicken gut microbiota to design and development of new therapeutic strategies for prevention and control of FLHS in laying hens.

## Data Availability

The data analyzed during the current study are available from the corresponding author on reasonable request.

## Abbreviations

ACOX1: Acyl-CoA oxidase 1
ALP: Alkaline phosphatase
ALT: Alanine transaminase
AST: Aspartate transaminase
ChREBP1: Carbohydrate response element-binding protein 1
CPT1: Carnitine palmitoyl transferase 1
FAS: Fatty acid synthase
FLHS: Fatty liver hemorrhagic syndrome
FMT: Fecal microbiota transplantation
FXR: Farnesoid X receptor
GBE: *Ginkgo biloba *extract
GLU: Glucose
GPAT1: Glycerol-3-phosphate acyltransferase 1
GSH: Glutathione
GSH-Px: Glutathione peroxidase
HE: Hematoxylin–eosin staining
HFD: High-fat diet
HMGCR: 3-Hydroxy-3-methylglutaryl-CoA reductase
IL-6: Interleukin 6
LXRα: Liver X receptor alpha
L-FABP: Liver fatty acid binding protein
MAFLD: Metabolic dysfunction-associated fatty liver disease
MDA: Malondialdehyde
NAFLD: Non-alcoholic fatty liver disease
NAS: NAFLD activity score
NF-*κB*: Nuclear factor kappa-B
PPARα: Peroxisome proliferator-activated receptor alpha
PPARγ: Peroxisome proliferator-activated receptor gamma
ROS: Reactive oxygen species
RT-qPCR: Real-time quantitative polymerase chain reaction
SCD1: Stearoyl-CoA desaturase 1
SCFAs: Short-chain fatty acids
SREBP-1c: Sterol regulatory element-binding protein 1c
T-AOC: Total antioxidant capacity
TC: Total cholesterol
TG: Triglycerides
TLR4: Toll-like receptors 4
TNF-α: Tumor necrosis factor alpha
T-SOD: Superoxide dismutase
